# Treatment of Brachymetatarsia Involving the Great Toe

**DOI:** 10.2106/JBJS.OA.17.00046

**Published:** 2018-04-19

**Authors:** Hui Taek Kim, Sung Min Hong, In Hee Kim

**Affiliations:** 1 Department of Orthopaedic Surgery and Biomedical Research Institute, Pusan National University Hospital, Busan, South Korea

## Abstract

**Background:**

Brachymetatarsia is usually treated by lengthening the metatarsals, but excessive lengthening can be associated with complications. Our technique combines 1-stage step-cut lengthening of the first metatarsal with shortening and/or lengthening of the neighboring metatarsals and/or phalanges.

**Methods:**

Twenty-four feet (15 patients) were treated for first-ray brachymetatarsia. Widely available commercial image-editing software was used to make a preoperative plan for each patient, with emphasis on the creation of a cosmetically satisfying toe-length arc with minimum shortening and lengthening of the affected metatarsals and proximal phalanges. Length gain and percentage increase were also recorded postoperatively. The American Orthopaedic Foot & Ankle Society (AOFAS) hallux metatarsophalangeal-interphalangeal scoring system was used for clinical evaluation.

**Results:**

In all 24 feet, smooth parabolas were created at the level of the metatarsal heads and at the toe tips. All patients showed osseous union, and no complications were noted. However, most patients showed mildly restricted range of motion of the first metatarsophalangeal joint. The mean AOFAS score of the hallux significantly improved from 88.3 preoperatively to 98.1 at the latest follow-up (p < 0.001).

**Conclusions:**

One-stage step-cut lengthening of the first metatarsal combined with shortening and/or lengthening of the adjacent metatarsal and phalangeal bones provides excellent cosmetic and functional results.

**Level of Evidence:**

Therapeutic Level IV. See Instructions for Authors for a complete description of levels of evidence.

The treatment of brachymetatarsia by lengthening of the short digits is associated with many difficulties. Stiffness of the first metatarsophalangeal joint is the most common complication. Deformities such as hallux valgus and cavus deformity of the metatarsus often necessitate a secondary surgical procedure. In addition, callus fractures, pin breakage, and pin-site infection may occur when lengthening is attempted by distraction osteogenesis^1-6^.

Our technique includes acute 1-stage lengthening and internal fixation of the first metatarsal and requires careful preoperative planning. We perform combined shortening and/or lengthening of adjacent bones as needed to avoid excessive first ray lengthening, which may lead to complications.

## Materials and Methods

Twenty-four feet (15 patients) with first-ray brachymetatarsia were studied; 9 patients had bilateral involvement (Table I). The study group included 13 females and 2 males with a mean age of 15.9 years (range, 11 to 26 years) at the time of the procedure. The mean duration of follow-up was 4.7 years (range, 1.7 to 12.1 years). In all patients, the first metatarsal was noticeably shorter in length than the second and the difference in length was not due to trauma. One absolute indication for surgical intervention was pain that was secondary to deformities (e.g., cock-up toe, claw toe), calluses, transfer lesions, or metatarsalgia associated with wearing shoes. Nine patients reported pain and discomfort in the sole and toes as a result of excessive pressure on the longer metatarsal heads as well as crowding of the normal-length toes over the short toes within shoes when walking. Cosmesis was very important to all patients, especially the teenage patients, who were extremely self-conscious about wearing open-toed shoes or going barefoot. Five patients had a family history of brachymetatarsia among their siblings and/or parents. There were no other associated malformations in the patients or other family members.

**TABLE I:** Data on the Patients*

										AOFAS Score for First Ray *(points)*			
		Affected Toe, Gain in Length (Percentage Increase)		Shortening of Neighboring Metatarsals or Phalanges				Total ROM of Hallux MTP Joint at Latest Follow-up *(°)*		Preop.		Latest Follow-up	
Case	Sex, Age *(yr)*	R	L	R	L	Bone Graft(s) in the Lengthened Metatarsal	Follow-up *(yr)*	R	L	R	L	R	L
Only the first ray involved: treated with Z-lengthening (7 feet in 5 patients)													
1	F, 13	First, 8 mm (23%)	First, 12 mm (38%)	—	—	Hydroxyapatite (bilateral)	2.3	100	100	89	82	100	97
2	M, 14	—	First, 11 mm (32%)	—	—	Hydroxyapatite	1.8	—	85	—	100	—	100
3	F, 11	First, 16 mm (38%)	First, 14 mm (37%)	—	—	—	2.0	70	80	89	88	97	100
4	F, 13	—	First, 14 mm (36%)	—	—	—	2.2	—	95	—	88	—	100
5	M, 12	First, 6 mm (12%)	—	—	—	—	3.0	115	—	88	—	97	—
First and other rays involved: first metatarsal treated with Z-lengthening, other metatarsals lengthened or shortened (11 feet in 7 patients)													
6	F, 12	First, 8 mm (25%); fourth, 13 mm (28%)	—	—	—	Iliac bone grafted into fourth metatarsal	2.3	95	—	87	—	97	—
7	F, 14	First, 10 mm (31%); third, 12 mm (29%); fourth, 13 mm (31%)	First, 11 mm (34%); third, 9 mm (24%); fourth, 8 mm (20%)	—	—	Iliac bone grafted into third, fourth metatarsals	1.7	95	80	92	90	95	100
8	F, 13	First, 8 mm (26%); fourth, 8 mm (17%)	First, 7 mm (20%); fourth, 7 mm (14%)	—	—	Iliac bone grafted into fourth metatarsal	3.1	100	110	90	87	100	97
9	F, 26	—	First, 14 mm (38%); fourth, 18 mm (41%)	—	Metatarsal: second, 3 mm; third, 3 mm; fifth, 8 mm	Excised fragments from second, third metatarsals to fourth, and from fifth metatarsal to first	4.6	—	110	—	83	—	97
10	F, 16	—	First, 14 mm (37%); third, 13 mm (36%)	—	Metatarsal: second, 9 mm	Excised fragment from second metatarsal grafted into third metatarsal	8.8	—	105	—	88	—	97
11	F, 24	First, 11 mm (31%)	First, 14 mm (40%); fourth, 7 mm (15%)	Metatarsal: second, 5 mm; third, 5 mm	Metatarsal: second, 5 mm; third, 6 mm	Excised fragments from second and third metatarsals to first, iliac bone to fourth	2.7	85	95	89	87	100	97
12	F, 19	First, 12 mm (34%); fourth, 8 mm (17%); fifth 12 mm (26%)	First, 11 mm (31%); fourth, 7 mm (15%); fifth, 6 mm (13%)	Metatarsal: second, 5 mm; third, 5 mm	Metatarsal: second, 6 mm; third, 4 mm	Iliac bone grafted into first metatarsal, excised fragments from second and third metatarsals grafted into fourth metatarsal	9.9	90	90	88	87	97	100
First and other rays involved: first ray not treated, other metatarsals and/or phalanges lengthened or shortened (6 feet in 3 patients)													
13	F, 13	First, none; fourth, 8 mm (17%)	First, none; fourth, 8 mm (18%)	Metatarsal: second, 7 mm; third, 7 mm	Metatarsal: second, 9 mm; third, 6 mm	—	6.0	—	—	90	87	97	97
14	F, 19	First, none; fourth, 12 mm (24%)	First, none; fourth, 14 mm (26%)	—	—	Iliac bone grafted into fourth metatarsal of both feet	8.0	—	—	89	85	100	97
15	F, 19	First, none; fourth, 14 mm (29%)	First, none; fourth, 15 mm (29%)	Metatarsal: second, 9 mm; third, 9 mm; Proximal phalanx: fifth, 5 mm	Metatarsal: second, 9 mm; third, 9 mm; Proximal phalanx: fifth, 6 mm	Iliac bone grafted into to both fourth metatarsals	12.1	—	—	89	87	95	100

* Total ROM of MTP = sum of extension and flexion (range of motion) of metatarsophalangeal joint. AOFAS = American Orthopaedic Foot & Ankle Society hallux metatarsophalangeal-interphalangeal scoring system.

The patients were clinically assessed according to the American Orthopaedic Foot & Ankle Society (AOFAS) hallux metatarsophalangeal-interphalangeal scoring system^7^, with the score being classified as excellent (>85 of 100 points), good (71 to 85 points), fair (56 to 70 points), or poor (<56 points). The mean preoperative AOFAS score for all patients was 88.3 points (range, 82 to 100 points). The Wilcoxon signed-rank test was used to compare preoperative and postoperative AOFAS scores, and the level of significance was set at p < 0.05. The range of motion of the metatarsophalangeal joint of the hallux at the time of the latest follow-up was classified as normal (no loss of motion), mild restriction (>75° of motion), moderate restriction (30° to 75° of motion), or marked restriction (<30° of motion)^7^.

### Preoperative Plan

Our method involves the use of widely available, commercial image-editing software (Adobe Photoshop and Microsoft PowerPoint) and preoperative anteroposterior radiographs of the foot (made with the patient bearing full weight with all toes fully straightened) that are stored in a picture archiving and communication system (PACS) (Marosis m-view 4.5; Marotech). This technique is easy to learn and is more accurate, more efficient, and clearer than using hand-made drawings, thus making the procedure itself easier to perform^8^ (Figs. 1-A, 1-B, and 1-C).

**Figs. 1-A, 1-B, and 1-C** Images created for use in the preoperative planning process.

**Fig. 1-A:**
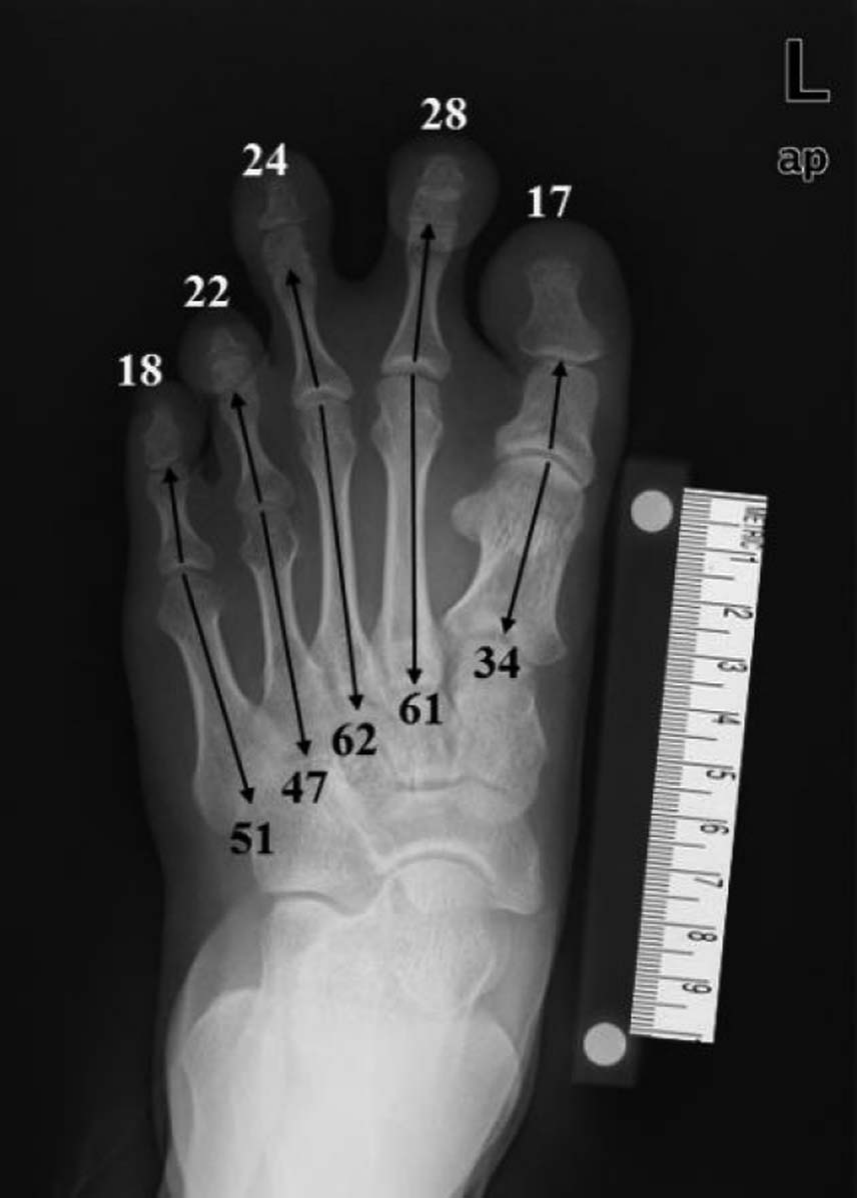
Anteroposterior radiograph of the foot, showing brachymetatarsia in the first, fourth, and fifth rays. The numerical values represent the lengths (in millimeters) of the metatarsals (black numbers) and the phalanges (white numbers).

**Fig. 1-B:**
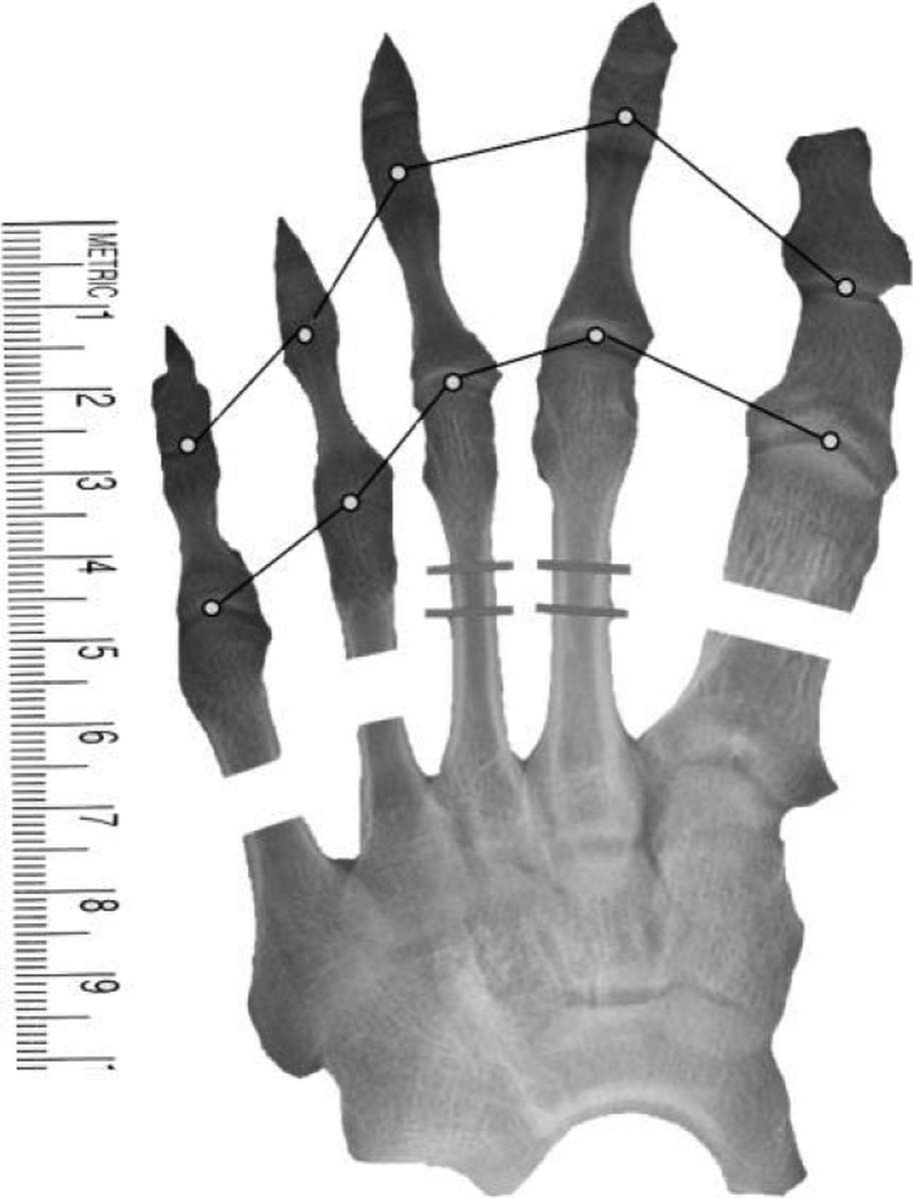
A half-completed image (created with Microsoft PowerPoint) for use in Step 6 of the preoperative plan, with the distal halves of the first, fourth, and fifth metatarsals moved outward.

**Fig. 1-C:**
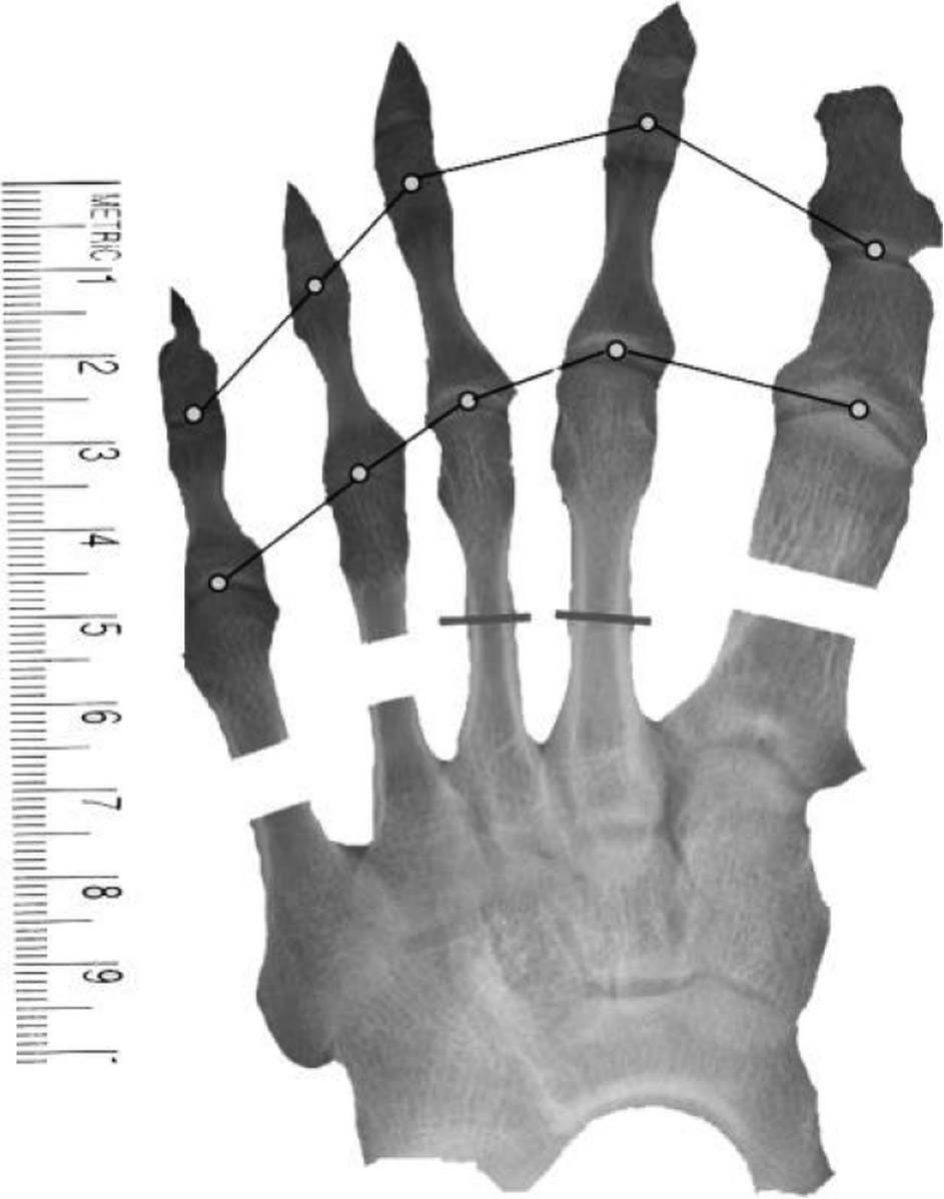
Image (created with Microsoft PowerPoint) showing shows the segments removed from the second and third metatarsals for later insertion into the fourth and fifth ray gaps, resulting in smooth curves at the metatarsal heads and at the toe tips.

**Fig. 2:**
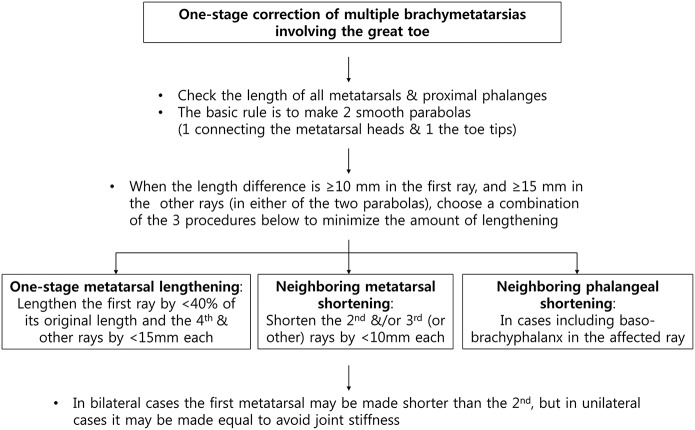
Decision tree for the 1-stage correction of multiple brachymetatarsia involving the great toe.

Our guiding concept is to make 2 smooth curves, 1 connecting the metatarsal heads and the other connecting the toe tips^9^, that minimize and equalize (as much as possible) the total amount of bone shortening and lengthening (Fig. 2). As the toes are often flexed in radiographs, the preoperative plan involves the creation of a line that connects the proximal interphalangeal joints (as described below). The toe-tip arc is also considered when performing the actual procedure.

### Preparation of Images (Figs. 1-A, 1-B, and 1-C)

*Step 1.* The anteroposterior radiograph of the affected foot was converted to a JPEG file by means of either scanning or digital photography. Radiographs that are stored in the PACS can be converted directly into JPEG files.

*Step 2.* The JPEG file was then opened in Adobe Photoshop, and the Magnetic Lasso or Polygonal Lasso Tool was used to precisely outline the whole image of the foot from the midfoot to the tips of the toes. The resulting cropped image was moved in Adobe Photoshop to a newly created layer and was saved as a separate JPEG file. The same procedure was performed for the whole first toe (from the metatarsal base to the distal phalanx), for each of the other 4 toes (also from the metatarsal base to the distal phalanx), and for the remaining part of the foot (from the midfoot to the base of the metatarsals). In each instance, the resulting images were saved as separate JPEG files that were available for repeated use. Next, a transverse, bisecting line was drawn across each metatarsal in the image. The distal half of each metatarsal that needed lengthening or shortening was then outlined and saved as its own JPEG file. The remaining portions (i.e., basal halves) of those metatarsals were also outlined, and the images were saved as their own JPEG files.

*Step 3.* All of the files (i.e., the whole image from the midfoot to the toe tips, the whole first ray bisected transversely through the metatarsal, any other metatarsals that were bisected transversely, the remaining part of the foot, a 10-cm template, a 10-cm ruler, and any plates that would be used for fixation after osteotomy) were then opened in Microsoft PowerPoint.

*Step 4.* The images of all transversely bisected metatarsals and of the remaining part of the foot were carefully overlaid atop the whole image from the midfoot to the toe tips by adjusting the size of the images, and the latter image was deleted, leaving only the overlays. A 10-cm template and a 10-cm ruler were placed in the corner of the resulting image. The ruler was fitted exactly to the size of the template.

*Step 5.* Line segments were drawn to connect the 5 metatarsal heads, and another set of line segments connecting the proximal interphalangeal joints was used to check for the presence of brachybasophalangia (i.e., a short proximal phalanx). The lengths of all metatarsals and proximal phalanges were measured accurately using the template and ruler.

*Step 6.* The images of the distal part of the affected rays (including the distal aspect of the metatarsal and the phalanges) were moved distally until the line segments that connected the metatarsal heads formed a visually satisfying, smooth curve (or parabola), and then the length of the empty spaces between the distal and proximal portions was measured, with this measurement corresponding to the amount of lengthening that would be needed. The goal was to create arcs that would achieve the best functional and cosmetic results and that would require the least lengthening of the affected metatarsals. In cases of multiple brachymetatarsia, the possibility of shortening the neighboring metatarsals or phalanges was evaluated first because, in normal feet, the length of the first metatarsal can be less than, equal to, or greater than that of the second metatarsal. Thus, the “optimal” or desired curve that connects the first metatarsal heads could be drawn differently for each patient, depending both on the amount of lengthening of the first and other rays and on whether the involvement was bilateral or unilateral. In bilateral cases, we thought it was better to place the first metatarsal head as shorter than the second in order to lessen the lengthening of the first metatarsal. In unilateral cases, we attempted to have the level of the great toe the same in both feet.

*Step 7.* The shortening of the metatarsals of unaffected rays was considered when the required lengthening of the first metatarsal exceeded 40% of the original length^10^ or 1.5 cm (although we now believe that 1.0 cm is a safer limit) because metatarsophalangeal joint stiffness can be severe beyond 1.0 cm.

*Step 8.* Shortening of the neighboring proximal phalanges was indicated when the curve that connected the tips of the toes was not satisfactory even after the creation of a smooth curve that connected the metatarsal heads. This problem occurred when brachybasophalangia was also present in the affected ray.

### Surgical Procedure for First Metatarsal Lengthening

**Fig. 3:**
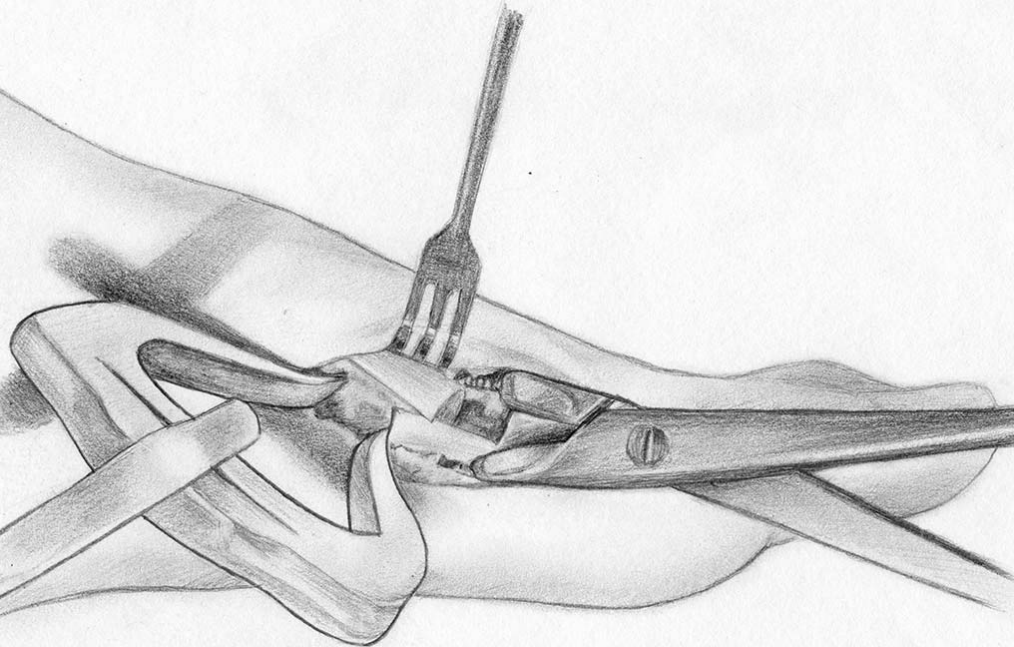
At the time of fixation of the plate, the lamina spreader is placed in the plantar side of the gap to maintain the required distance with slight extension of the distal fragment.

*Step 1.* The skin was opened on the medial side of the first metatarsal, and a step-cut osteotomy was made with use of a small electric saw at the medial side of the bone (Fig. 3). The placement of the 2 vertical half-cuts at the proximal and distal ends of the longitudinal osteotomy was decided after comparison with a small fixation plate, with the goal being to leave enough bone to allow for fixation with 2 screws at each end. After the 2 vertical half-cuts were carefully made, the cuts were longitudinally connected with use of the same small electric saw.

*Step 2.* The osteotomized first metatarsal was lengthened as planned while the metatarsophalangeal joint was dorsiflexed. Dorsiflexion became more difficult as the lengthening increased, and, when the joint could not be dorsiflexed at least 15° in the ankle-neutral position, the amount of the lengthening was reduced. The amount of lengthening also depended on the original length of the metatarsal because at least 0.5 cm of the 2 osteotomized bones had to overlap.

*Step 3.* A lamina spreader was used to longitudinally separate the 2 osteotomized osseous fragments. The lamina spreader was placed in the osteotomized gap, on either the plantar or dorsal side, and the gap was lengthened. However, at the time of fixation of the plate, the lamina spreader was placed in the plantar side of the gap to maintain the required distance with slight extension of the distal fragment (Fig. 3). It was considered important to prevent plantar flexion of the distal fragment at the time of fixation in order to avoid pain in the metatarsal head.

*Step 4.* With use of 2 small bone holders to hold the osteotomized proximal and distal bones vertically and horizontally where they overlapped, both the plantar flexion and valgus position of the metatarsal head were controlled until fixation was complete. A small plate (Leforte craniomaxillofacial system [Jeil Medical] or Leibinger Universal Mini-Plate [Stryker]) was placed on the bone while the lamina spreader and a small bone holder maintained the lengthened gap and the desired orientation of the distal fragment (slightly extended and without valgus angulation). Usually, we attempted to obtain 2-screw fixation in each end of the proximal and distal fragments, but when the metatarsal was too short for 2 screws to obtain purchase at each end, we placed 1 proximal screw into the medial cuneiform. When more stability was required, 1 or 2 screws were placed vertically through the overlapping part of the 2 osteotomized bones and/or a strut bone graft from the ilium was placed in the osteotomy gap.

*Step 5.* In the bone gap caused by lengthening, we used an interpositional bone graft from the ilium (2 feet), an excised metatarsal fragment from 1 or more neighboring digits (3 feet), or synthetic hydroxyapatite (OSG-DualPor; OssGen) (3 feet) in the first metatarsal (Table I).

### Surgical Procedure for Other Metatarsals and Toe Lengthening and/or Shortening

*Step 1.* The incision for the first metatarsal lengthening was medial. Any needed incisions in the second through fourth rays were dorsal. To minimize the number and length of the incisions on the foot dorsum for cosmesis, we found that it was possible to shorten the second and third metatarsals and to lengthen the fourth with use of a single dorsal incision over the third metatarsal (Figs. 4-A through 4-D). Lateral incisions were used for the fifth metatarsal.

**Figs. 4-A through 4-D** Case 12, a 19-year-old girl with brachymetatarsia in the first, fourth, and fifth rays in both feet. (The foot shown in Figures 1-A, 1-B, and 1-C is from the same patient.)

**Fig. 4-A:**
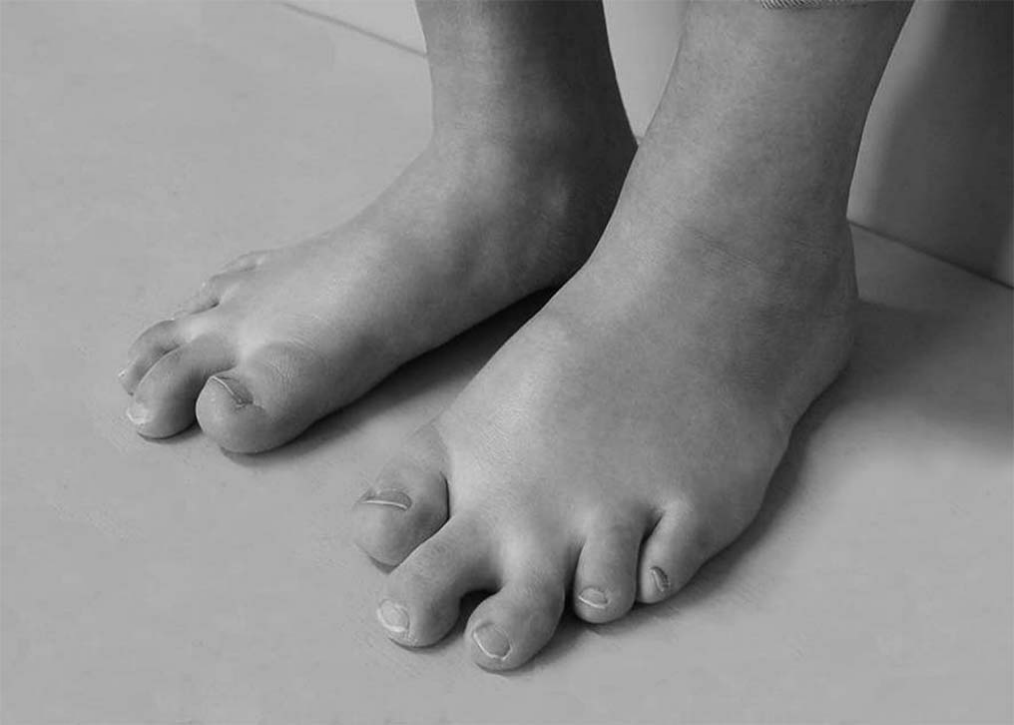
Preoperative photograph.

**Fig. 4-B:**
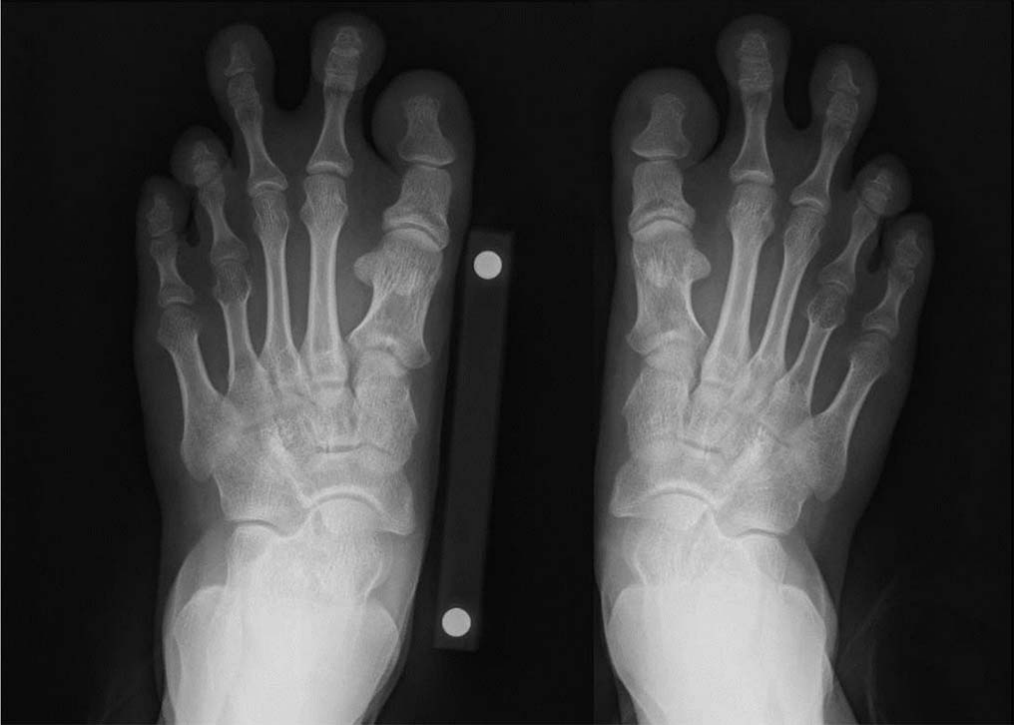
Preoperative anteroposterior radiograph of both feet, showing the short first, fourth, and fifth metatarsals, resulting in an irregular (non-smooth) curve (or parabola) that connects the 5 metatarsal heads.

**Fig. 4-C:**
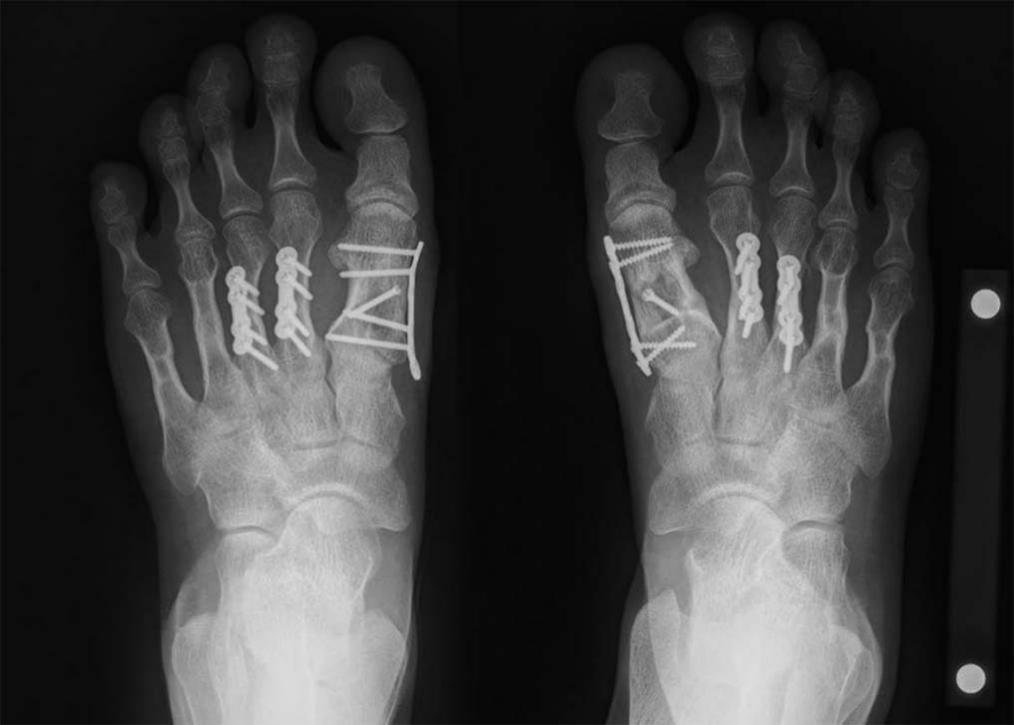
Radiograph made 1 year after 1-stage step-cut lengthening of the first metatarsal (which resulted in an increase of 12 mm [or 34% of the original length] in the right foot and 11 mm [31% of the original length] in the left foot), second and third metatarsal shortening (which resulted in 5 mm and 5 mm of shortening, respectively, in the right foot and 6 mm and 4 mm of shortening, respectively, in the left foot), and 1-stage lengthening of the fourth and fifth metatarsals with use of interpositional grafts from the second and third metatarsals that were fixed with intramedullary Kirschner wires.

**Fig. 4-D:**
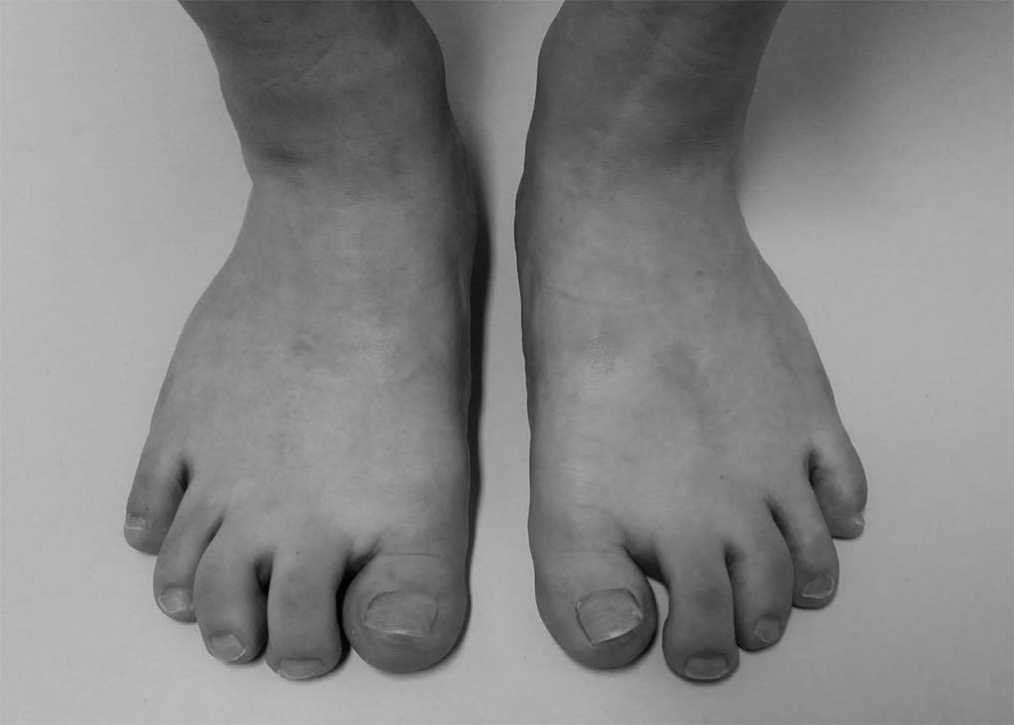
Photograph, made 9 years postoperatively, showing the improved cosmetic appearance of the feet. The medial and lateral incision scars are not noticeable. The second toe is slightly longer than the first (a “type-B” normal foot).

*Step 2.* Plate fixation was necessary to reduce the excised bone gap in the long second and third metatarsals; manual compression was maintained until plate fixation was completed. Two-screw fixation was sufficient in each end of the bone. Care was taken to make sure that the distal fragment was fixed evenly with the adjacent metatarsal heads and that it was not plantarly or dorsally flexed.

*Step 3.* When we placed the bone from the long metatarsal into the short fourth and/or fifth metatarsal, we used an intramedullary Kirschner wire (0.16 cm) instead of a plate, with the wire extending from the toe tip through the metatarsophalangeal joint and the osteotomized metatarsal.

*Step 4.* Although a short phalanx (i.e., basobrachyphalanx) with a short metatarsal was not often encountered, it was necessary to check for this possibility before the procedure. In such cases, lengthening the metatarsal too much in an attempt to achieve a smooth toe-tip curve might have caused the protruding metatarsal head to disrupt the metatarsal curve, possibly causing pain during walking. In such a case (e.g., Case 15), 1 or more neighboring phalanges had to be shortened to make a smooth toe-tip curve with minimum lengthening of the metatarsal (Table I).

*Step 5.* In the lengthened gap of the third, fourth, and fifth metatarsals, we used iliac bone and excised metatarsals, rather than synthetic hydroxyapatite, in order to ensure strong osseous union (Table I).

### Soft-Tissue Procedures

*Step 1.* For the first metatarsal, no soft-tissue procedures were performed because the amount of lengthening was minimized.

*Step 2.* For the other toes, Z-lengthening of the extensor, long and short flexor tendons, and plantar plate capsulotomy were frequently required to ensure a natural appearance without overly extending or flexing any toes. The goal was to create metatarsophalangeal joints that were flexible with full range of motion.

### After Treatment

All patients were managed with a short-leg (boot-style) cast for 6 weeks; partial weight-bearing on the heel was permitted during this time.

## Results

In all patients, smooth curves were restored at the levels of the metatarsal heads and toe tips. All patients had osseous union and a satisfactory clinical result, and all were satisfied with the cosmetic improvement. No complications (e.g., donor-site morbidity, neurovascular impairment, or length gains that were smaller than expected) were reported. The mean AOFAS score for the hallux significantly improved from 88.3 (range, 82 to 100) preoperatively to 98.1 (range, 95 to 100) at the time of the latest follow-up (p < 0.001), and all metatarsals received an excellent AOFAS score (>85 points).

In all 18 feet with a lengthened first metatarsal, the mean absolute length gain was 11.2 mm (range, 6 to 16 mm), and the mean percentage increase was 31.3% (range, 12% to 40%) (Table I). The mean metatarsophalangeal joint range of motion (dorsiflexion plus plantar flexion) was 94.4° (range, 70° to 115°) at the time of the latest follow-up. One ray had a normal range of motion of the metatarsophalangeal joint, 16 had mild restriction, and 1 had moderate restriction.

Bone shortening was carried out in 2 proximal phalanges and 20 metatarsals in the neighboring rays (Cases 9 through 13 and Case 15). The mean amount of bone shortening was 6.4 mm (range, 3 to 9 mm).

## Discussion

The 2 most widely used techniques for the treatment of brachymetatarsia are 1-stage lengthening with an interpositional bone graft and gradual lengthening by means of callotasis. The advantages and disadvantages of these techniques have been well described^11-13^. With both techniques, the problems are mainly related to excessive lengthening, which can result in stiffness or subluxation of the metatarsophalangeal joint and/or cavus or plantar angulation deformity of the distal fragment.

In our earlier attempts to treat brachymetatarsia, we gradually lengthened all short metatarsals with use of external fixation and noted the same complications that others have reported, such as metatarsophalangeal joint stiffness and subluxation^1-6,12-14^, hallux valgus^5,12,13^, malalignment (angulation)^3,5,6,13,14^, failure of bone formation^1,5,12-14^, and premature healing^4-6,13,14^. Our current approach for treating first-ray brachymetatarsia was first developed after we found that lengthening of up to 1.0 cm is not difficult and that metatarsophalangeal joint stiffness occurs beyond 1.0 cm of lengthening. We found metal plates that were strong enough to maintain the distracted portion and also learned how to prevent plantar flexion of the osteotomized distal fragment. Our current approach (Fig. 2) has been associated with a high rate of patient satisfaction because we do not lengthen the first toe too much and we also employ lengthening and/or shortening of the other toes. Lee et al., in a study in which 32 great toes underwent gradual distraction for the treatment of brachymetatarsia, reported 26 complications, most commonly metatarsophalangeal joint stiffness^15^. The mean length gains and mean percentage increase reported by those authors were considerably higher than the 11.2 mm and 31.3% in the present study, and the rate of complications in that study was also greater than that in the present study.

Our technique involving Adobe Photoshop and Microsoft PowerPoint takes time to learn, but, once learned, it provides surgical plans that are accurate in terms of both length and angular correction. We estimate that, with experience, it takes about 40 minutes to create a workable preoperative plan for each patient.

The relationship between transfer metatarsalgia in the second metatarsal head and postoperative shortening of the first metatarsal has been considered by several authors. Carr and Boyd^16^ proposed that 4 mm is an acceptable limit of postoperative shortening, and Schemitsch and Horne^17^ concluded that a postoperative first-to-second metatarsal length ratio of <0.825 was important. Singh and Dudkiewicz^18^ found that at least 8 mm (preferably 1.0 cm) of lengthening was needed to provide adequate relief of metatarsalgia. Regarding the normal curves of the metatarsal heads, we agree that there are 3 different types of curves (all normal) with different relationships between the first and second toes^19,20^. A type-B curve is one in which the first toe tip is shorter than the second. We prefer to attempt to achieve type-B curves in patients with bilateral involvement in order to minimize the lengthening of the first metatarsal. A type-B curve would be difficult to achieve in patients with unilateral involvement; in such patients, a type-A curve (with the first and second toes being equal in length) would be a better goal than a type-C curve (with the first toe being longer than the second), especially when the amount of lengthening required in the first metatarsal is >1.5 cm.

The best time for a surgical procedure for brachymetatarsia is when the growth plates of the metatarsals are closed, although we also perform the operation when the growth plates of the metatarsals and phalanges are near closure.

When we asked our patients with unilateral involvement about the asymmetrical foot lengths that would result from shortening the longer toes, all responded that it would not be a problem as the most conspicuous toe (the great toe) would be lengthened and the weight would be evenly distributed.

Our technique has limitations. First, it requires some time to learn, especially the creation of cosmetically acceptable curves that both minimize and equalize the total amount of shortening and lengthening. Second, 1-stage lengthening is not appropriate for patients requiring a >40% length increase in the first metatarsal or a >1.5-cm increase in any toe; for such patients, gradual lengthening should be adopted, although joint stiffness would then be a problem. We did not include scores for the second through fifth rays in the present study because of the great variety of initial conditions with which our patients presented.

In conclusion, careful creation of a preoperative plan that includes 1-stage Z-lengthening of the first metatarsal plus lengthening and/or shortening of adjacent metatarsals and phalanges provides good functional and cosmetic outcomes with a low rate of complications.
